# Concurrent Chemoradiotherapy With Nedaplatin Versus Cisplatin in Patients With Stage IIB-IVA Cervical Cancer: A Randomized Phase III Trial

**DOI:** 10.3389/fonc.2021.798617

**Published:** 2022-02-02

**Authors:** Shasha He, Yan Wang, Yulin Lai, Xinping Cao, Yufeng Ren, Yong Chen

**Affiliations:** ^1^ Department of Radiation Oncology, The First Affiliated Hospital, Sun Yat-sen University, Guangzhou, China; ^2^ Department of Radiation Oncology, Sun Yat-sen University Cancer Center (SYSUCC), Guangzhou, China

**Keywords:** cervical carcinoma, nedaplatin, cisplatin, toxicity, survival

## Abstract

**Background:**

In this trial, we aimed to assess the efficacy and safety of radiotherapy with nedaplatin or cisplatin in patients with locally advanced cervical cancer.

**Methods:**

We conducted an open-label, non-inferiority, phase III, randomized, controlled trial. Eligible patients with stage IIB-IVA cervical carcinoma were randomly assigned to receive either nedaplatin or cisplatin for two cycles concurrently with radiotherapy. We reported the therapy-associated harms and survival. The study was registered with chictr.org.cn, number ChiCTR1800020527.

**Results:**

We randomly assigned 68 patients to nedaplatin-based or cisplatin-based concurrent chemoradiotherapy. Study treatment was stopped early after a data analysis found a higher number of patients suffered severe hematologic harms in the nedaplatin group than in the cisplatin group. Patients in the nedaplatin group had a significantly higher frequency of grade 3-4 neutropenia (19·4% vs. 13%; *P* < 0·001), severe thrombocytopenia (16·1% vs. 4·3%), and grade 1-2 anemia (51·6% vs. 43·5%) than patients in the cisplatin group. The 1-year PFS and OS in the nedaplatin and cisplatin groups were similar.

**Conclusion:**

Our findings showed that nedaplatin-based concurrent chemoradiotherapy expressed remarkably higher severe hematologic harms which were mortal. Though the results were negative, the experiences and lessons we learned from it were important.

## Introduction

Cervical cancer is one of the most common malignant carcinomas in women, is currently the fourth leading cancer in women worldwide, and resulted in approximately 570 000 cases of cervical cancer and 311 000 deaths from the disease in 2018. The estimated age-standardized incidence of cervical cancer is 13·1 per 100 000 women globally and varies widely among countries (1). Compared with 40 years ago, the morbidity of cervical cancer has decreased due to the established screening program and available vaccines for human papilloma virus (HPV) in many countries, however, advanced cervical cancer cases remain prevalent, especially in developing countries (2). Currently the international standard treatment for advanced cervical cancer is platinum-based chemoradiotherapy (3). But the 5 year survival rate is still very low, for FIGO stage II, III, and IVA, the survival rate is 55%, 35%, and 15% respectively, and for locally advanced cases, the local recurrence rate is around 64% (4). With the development of intensity-modulated radiotherapy (IMRT) and improvement of radiotherapy accuracy, treatment effect for cervical cancer has also improved. While the main direction of current studies in concurrent chemoradiotherapy (CCRT) is still trying to improve efficacy and reduce adverse events.

Based on the findings of several random trials, the standard therapy for cervical carcinoma recommended by NCCN guidelines is platinum-based concurrent chemoradiotherapy; the most recommended is cisplatin (5, 6). However, due to the potential toxicity frequently seen in the gastrointestinal tract and kidney, clinical use of cisplatin may be restricted. In chemotherapy for non-small cell lung cancer (NSCLC), head and neck cancer (HNC), and cervical cancer, nedaplatin, as a derivative of cisplatin, is now frequently implemented as a new choice. Compared with cisplatin, nedaplatin seems to be less toxic to the gastrointestinal tract and kidney. Currently a phase III clinical trial proved that nedaplatin can be a new alternative to treat advanced or relapsed squamous cell lung carcinoma, with higher overall survival and lower toxicity when in combination with docetaxel compared to cisplatin (7). Besides, a recent non-inferiority randomized phase III trial compared the efficacy of nedaplatin against cisplatin in CCRT for stage II-IVB nasopharyngeal carcinoma. The study included 402 patients. The results showed the 2-year PFS in the cisplatin group was 89·9% vs. 88·0% in the nedaplatin group, with no statistic difference; the results were similar for overall survival (OS) and local relapse-free survival (LRFS). As for grade 3-4 toxicity, frequency of vomiting, hypopotassemia, and hyponatremia was significantly higher in the cisplatin group than nedaplatin group, whereas thrombocytopenia incidence was higher in the nedaplatin group. Toxicity events like allergy and rashes, constipation, nausea, hiccup, ototoxicity, and renal toxicity were also higher in the cisplatin group. As concluded by this study, nedaplatin had comparable efficacy but lower toxicity, presenting better life quality to the patients. So again, nedaplatin was proved to be a possible alternative to cisplatin in chemotherapy (8).

However, there is only one retrospective study comparing the efficacy and safety of nedaplatin against cisplatin in cervical cancer treatment (9). Compared with cisplatin, nedaplatin treatment expressed a higher relapse rate, lower overall survival, but non-significant difference in grade 3-4 toxicities. According to this observation, nedaplatin was not supported as an alternative to cisplatin in chemotherapy. While due to the limitations of case selection bias of the retrospective study as well as the absent proof from prospective studies, more efforts are needed to verify the alternative role of nedaplatin in cervical cancer. We tried to initiate a prospective, randomized, and controlled study and evaluate the PFS and toxicities of nedaplatin against cisplatin in combination with IMRT as CCRT to advanced-stage cervical cancer patients.

## Methods

### Study Design and Participants

The open-label, non-inferiority, randomized, controlled trials in patients with cervical carcinoma were designed in China. The study was registered with chictr.org.cn, number ChiCTR1800020527. Eligible patients were aged between 18 and 65 years and had histologically confirmed squamous cell carcinoma, adenocarcinoma, or adenosquamous carcinoma; a clinical stage of IIB-IVA (according to the FIGO 2018 staging system); no evidence of distant metastasis assessed by imaging evaluation (chest film or CT, or abdominal ultrasonography or CT, or bone scan, or PET-CT); adequate hematological function (white blood cell count ≥4000 per μL, platelet count ≥100000 per μL, and hemoglobin ≥90 g/L); adequate renal function (creatinine clearance ≥60 mL/min); adequate hepatic function (serum bilirubin, alanine aminotransferase, and aspartate aminotransferase ≤2·0 times the upper limit of normal); and a performance status as per the Karnofsky score of at least 70. The exclusion criteria included previous radiotherapy or chemotherapy for cervical carcinoma; the presence of relapse or distant metastasis; a previous malignancy (apart from carcinoma *in situ* of the cervix, or basal or squamous cell carcinoma of the skin); the presence of uncontrolled life-threatening illness; and pregnancy or lactation. Other key exclusion criteria were any mental disorder or somatic comorbidities of clinical concern. The study protocol was approved by the ethics committee or institutional review board at our center, and all patients provided written informed consent.

### Sample Size Evaluation

The sample size was calculated by a professional statistician using the Power and Sample Size Program software. PFS at 2 years was assumed to be 65% in both groups, and the non-inferiority margin was set as 10%, based on survival data reported in the studies investigating the effect of adjuvant chemotherapy in advanced stage cervical cancer. Thus, to show non-inferiority, the upper limit of the 95% CI for the difference in 2-year PFS between the two groups (cisplatin group minus nedaplatin group) could not exceed 10%. With 85% power and a one-sided type I error of 2.5%, enrollment over 2 years, and a follow-up of 5 years, we needed at least 676 patients (338 in each group) to allow for a 10% dropout.

### Randomization and Masking

Eligible patients were randomly assigned (1:1) to receive either nedaplatin-based or cisplatin-based concurrent chemoradiotherapy. Random assignment was done by a computer-generated random number code at the Clinical Trials Centre. Details of the random allocations were contained in sequentially numbered, opaque, sealed envelopes prepared by a statistician (QL), who was also involved in the statistical analysis and interpretation and the toxicity and data review, with the consideration of stratification factors, block, sequential number, and opacity. After informed consent was obtained from eligible patients, the investigators opened the envelopes sequentially and allocated patients to the corresponding interventions.

### Procedures

Pretreatment assessment consisted of a complete physical examination, pathological examination, enhanced MRI or enhanced CT of the pelvic cavity (CT was indicated only in patients with contraindication to MRI), chest scan (radiograph or CT), liver scan (abdominal sonography or CT), electrocardiography, bone scan, complete blood count with differential count, biochemical profile, and tumor biomarker. Whole body ¹⁸F-fluorodeoxyglucose (¹⁸F-FDG) PET-CT was optional and was performed at the discretion of the attending physician.

Patients assigned to the nedaplatin group received 80-100 mg/m² of nedaplatin as a 2 h intravenous infusion on days 1 and 22 for two cycles concurrently with radiotherapy, and patients assigned to the cisplatin group received 80-100 mg/m² of cisplatin as a 4 h intravenous infusion on days 1 and 22 for two cycles concurrently with radiotherapy. Because chemotherapy might reduce the incidence of distant metastasis, if only one cycle of concurrent chemotherapy was completed during the radiotherapy phase, then the second cycle of chemotherapy was given within 1 week after completion of radiotherapy as planned. If the second dose of chemotherapy was not administered in this time, it was not given. To prevent the nephrotoxicity of cisplatin, the cisplatin was administrated on days 1–3 with a total dose of 80-100mg/m^2^. External beam radiation therapy (EBRT) in this study was administrated with five daily fractions per week for 5 weeks. A planning target volume was created by adding a three-dimensional margin of 5 mm to the delineated target volume to compensate for the uncertainties in treatment set-up and internal organ motion. The gross target volume of the primary tumor in cervix (GTVp): primary tumor volume in the cervical and invasion area was determined by clinical examination and imaging. GTVp1 included the parametrium. The lymph nodes target volume in the pelvic cavity (GTVnd): the volume of metastatic lymph nodes in the pelvic was observed clinically or by imaging (Criteria for imaging diagnosis: ① short axis ≥1cm; ② necrotic foci in the center; ③ extracapsular invasion; ④ short axis in clusters ≥0·8 cm). Clinical target volume (CTV) was defined as the paravaginal, paracervical, parauterus, obturator lymphatic drainage area, internal iliac lymphatic drainage area, external iliac lymphatic drainage area, 1-3 sacrum lymphatic drainage area, and lymphatic drainage area of common iliac artery. The prescribed dose was 60 Gy in 25 fractions for the planning target volumes derived from GTVp1 and GTVnd; 45-50 Gy in 25 fractions for the planning target volumes derived from CTV. EBRT was followed by image-guided brachytherapy (BT) of 28Gy/4 fractions or 30Gy/5 fractions, once or twice a week.

The National Cancer Institute Common Toxicity Criteria version 5·0 scale was used to assess chemotherapy and acute radiation toxicity. Chemotherapy dose adjustments were allowed for subjective/objective evidence of developing hematological or non-hematological toxicity during treatment. In case of hematological toxicity, chemotherapy was withheld until the nadir values were 1500 cells per μL or higher for the absolute neutrophil count and 100 000 per μL or higher for the platelet count. For renal toxicity, chemotherapy was withheld until creatinine clearance was higher than or equal to 60 mL/min. Dose modifications of nedaplatin or cisplatin were intended to be permanent for hematological and non-hematological toxicity (i.e., if a patient’s dose was reduced to 80 mg/m², it remained at the reduced dose for the duration of their treatment), and based on the nadir blood counts and interim toxicities of the preceding cycle. Modifications in the dose of nedaplatin or cisplatin were planned for neutropenia, thrombocytopenia, or neurotoxicity.

### Outcomes

The primary endpoint was therapy-associated toxicity, the prespecified secondary endpoints were progression-free survival and overall survival at 1 year. Progression-free survival was defined as the time from random assignment to documented local or regional relapse, distant metastasis, or death from any cause, whichever occurred first. Overall survival was defined as the time from random assignment to death from any cause or censored at the date of last follow-up.

The proportion of patients who had a complete response was defined as those with all pathological cervical lymph nodes of less than 10 mm in the short axis and no unequivocal soft tissue mass in the local region. The proportion of patients who had a partial response was defined as those who had at least a 30% decrease in the sum of diameters of all target lesions, taking the baseline sum diameters as a reference. The proportion of patients who suffered a progressive disease was defined as those who had at least a 20% increase in the sum of diameters of all target lesions, taking the baseline sum diameters as a reference; or a new target lesion was detected. The proportion of patients who remained with stable disease was defined as those who did not reach the level of partial response or progressive disease, taking the baseline sum diameters as a reference.

### Statistical Analysis

Analyses were done with SPSS 22·0. We used χ^2^ test or Fisher’s exact test for categorical variables and Mann-Whitney U tests for continuous variables to assess the differences between groups. Adverse events were compared by χ^2^ test. Survival outcomes were estimated using the Kaplan–Meier method and compared with log-rank test. The statistical test was two-sided, and *P* < 0·05 was considered significant.

## Results

### Patients and Treatment

**Figure 1:**
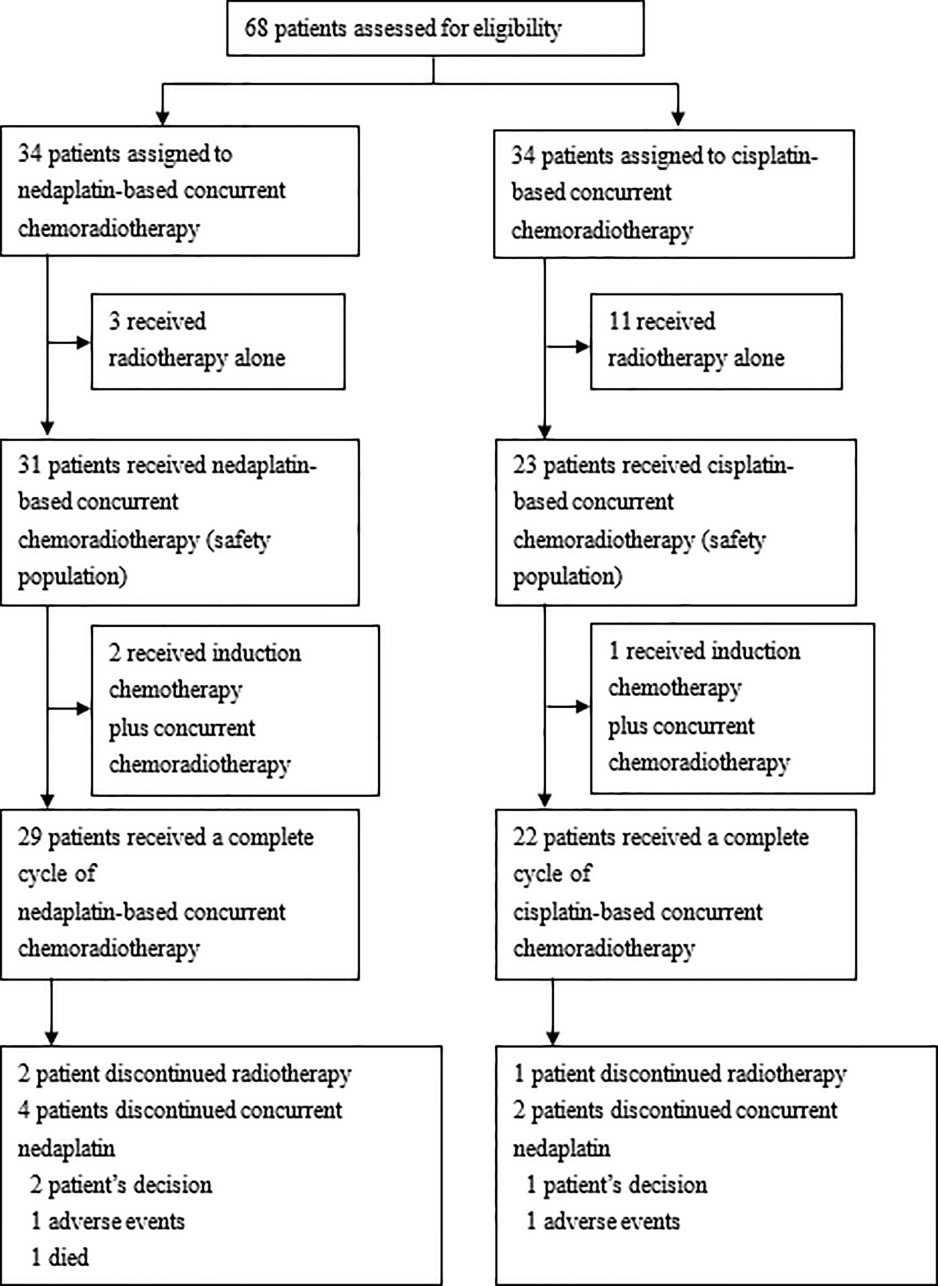
Trial profile.

Between September 2018 and June 2019, the last date of terminating the trials, we randomly assigned 68 patients to nedaplatin-based (n=34) or cisplatin-based (n=34) concurrent chemoradiotherapy in the CCRT group of patients with locally advanced cervical cancer for eligibility. We excluded patients who received radiotherapy only, 31 patients received nedaplatin-based concurrent chemoradiotherapy and 23 patients received cisplatin-based therapy. Of the 68 patients who had been randomly assigned to undergo concurrent chemoradiotherapy, 54 (79.4%) started protocol-defined therapy and were included in the safety population. A total of 3 patients in the nedaplatin group and 11 patients in the cisplatin group withdrew from the trial before the initiation of trial treatment (received chemoradiotherapy alone). A total of 29 patients received a completed cycle of nedaplatin-based concurrent chemoradiotherapy; 4 patients did not complete all two cycles (the reasons included two patients declining the treatment, one discontinuation due to adverse events, one died); 22 patients received a completed cycle of cisplatin-based concurrent chemoradiotherapy; 2 patients did not complete all two cycles (the reasons included one patient declining the treatment, one discontinuation due to adverse events) (**Figure 1**).

Baseline demographic and clinical characteristics are presented in **Table 1** and **Appendix p1**, including age and overall stage of cancer which were balanced between the two treatment groups. Most patients were stage II-III and most patients received a 80 mg/m^2^ dose of concurrent chemotherapy drugs. Overall, two patients died and three suffered distant metastasis in the nedaplatin group; one patient died in the cisplatin group.

**Table 1:** Baseline characteristics of all 68 patients in the intention-to-treat analysis.

Variable	N (%)	Nedaplatin group	Cisplatin group	*P*
Total	68 (100%)	34 (100%)	34 (100%)	
Age (years)				0·967
< 54	35 (51·5)	18 (52·9)	17 (50·0)	
≥ 54	33 (48·5)	16 (47·1)	17 (50·0)	
Stage (FIGO 2018)				0·559
II	37 (54·4)	18 (52·9)	19 (55·9)	
III	29 (42·6)	14 (41·2)	15 (44·1)	
IVA	2 (4·0)	2 (5·9)	0 (0)	
Dose of concurrent chemotherapy				
80 mg/m^2^	47	26	21	
100 mg/m^2^	7	5	2	
Death	3	2	1	
Distant metastasis	3	3	0	

### Efficacy

The tumor response were evaluated by pelvis magnetic resonance imaging. Overall, after concurrent chemoradiotherapy, in the nedaplatin group, 79·4% of the patients (27 of 34) had a complete response, four (11·8%) had a partial response, two (5·9%) remained stable, and one (2·9%) suffered progression. In the cisplatin group, 73·6% of the patients (25 of 34) had a complete response, five (14·7%) had a partial response, three (8·8%) remained stable, and one (2·9%) suffered progression. Tumor response rate were also evaluated 3 months after the whole therapy. In the nedaplatin group, 85·3% of the patients (29 of 34) had a complete response, one (5·9%) had a partial response, two (5·9%) remained stable, and two (5·9%) could not be assessed. In the cisplatin group, 82·3% of the patients (28 of 34) had a complete response, two (5·9%) had a partial response, two (5·9%) remained stable, and two (5·9%) could not be assessed. During follow-up, two patients died, one died of tumor distant failure and another died of severe therapy-associated harms. Three suffered distant metastasis in the nedaplatin group, including the sites of lung, bone, and lymph nodes. In the cisplatin group, one patients died of tumor and none suffered metastasis (**Table 2** and **Appendix p2**).

**Table 2:** Survival and response to treatment.

Variable	Nedaplatin group N=34	Cisplatin group N=34
Progression-free survival		
Recurrence, distant metastasis, or death — no. (%)	5 (14·7%)	1 (2·9%)
Overall survival		
Death — no. (%)	2/34 (5·9%)	1 (2·9%)
Response to concurrent chemoradiotherapy		
Complete response — no./total no. (%)	27/34 (79·4%)	25/34 (73·6%)
Partial response — no./total no. (%)	4/34 (11·8%)	5/34 (14·7%)
Stable disease — no./total no. (%)	2/34 (5·9%)	3/34 (8·8%)
Progressive disease — no./total no. (%)	1/34 (2·9%)	1/34 (2·9%)
Response to whole treatment		
Complete response — no. (%)	29/34 (85·3%)	28/34 (82·3%)
Partial response — no. (%)	1/34 (2·9%)	2/34 (5·9%)
Stable disease — no. (%)	2/34 (5·9%)	2/34 (5·9%)
Could not be assessed — no. (%)	2/34 (5·9%)	2/34 (2·9%)

**Figure 2:**
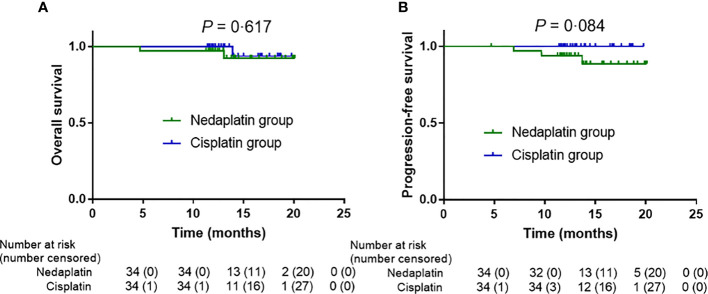
In the intention-to-treat analysis, survival outcome differences in the nedaplatin and cisplatin groups. Kaplan–Meier overall survival **(A)**, progression-free survival **(B)**.

The data cutoff date for the analyses was 4 June 2020. The median follow-up time for overall survival was 13·87 months (IQR 12·20–16·52) for the intention-to-treat analysis. Overall survival at 1 year was 97·1% in the nedaplatin group and 100% in the cisplatin group (log-rank, *P* = 0·617; **Figure 2A**); progression-free survival was 93·9% and 100%, respectively (log-rank, *P* = 0·084; **Figure 2B**). In the per-protocol analysis, the median follow-up time for overall survival was 13.87 months (IQR 12·12–17·37). One-year overall survival was 96·8% in the nedaplatin group and 100% in the cisplatin group (log-rank *P *= 0·779; **Appendix p3A**); progression-free survival was 93·3% and 100% (log-rank, *P* = 0·134; **Appendix p3B**).

### Treatment-Related Toxicities

The safety population included 31 patients in the nedaplatin group and 23 patients in the cisplatin group, who were treated with both chemotherapy and radiation treatment. In total, over half of patients suffered grade 1-2 neutropenia, and grade 3-4 severe neutropenia were seen in over 10% of patients. We recorded a higher frequency of grade 3-4 neutropenia in the nedaplatin group than the cisplatin group (19·4% vs. 13%; *P* < 0·001). Among that, two patients suffered febrile neutropenia and one went through neutropenic infection in the nedaplatin group. Also, grade 1-2 anemia (51·6% vs. 43·5%; *P* = 0·003) and thrombocytopenia (*P* = 0·001) in the nedaplatin group were more prevalent than in the cisplatin group. Whereas grade 1-2 nausea (69·6% vs. 61·3%; *P* = 0·029) and grade 3-4 nausea (26·1% vs. 9·7%; *P* < 0·001) in the cisplatin group was higher than in the nedaplatin group. Besides, more patients were found to have mild hypokalemia in the cisplatin group (26·1% vs. 13·0%; *P* = 0·034). The frequencies of adverse events of vomiting, diarrhea, constipation, weight loss, fatigue, pain, dermatitis, urocystitis, enteritis, hepatotoxic and nephrotoxic events, and allergic reaction did not differ between the two treatment groups (**Table 3**).

**Table 3:** Adverse events during chemoradiotherapy in the safety population.

Events	Nedaplatin group (n=31)			Cisplatin group (n=23)			*P* value for events grade 1-2	*P* value for events grade 3-4
	Grade1-2n (%)	Grade3-4n (%)	Grade1-4n (%)	Grade1-2n (%)	Grade3-4n (%)	Grade1-4n (%)		
Hematological								
Neutropenia	22 (71·0)	6 (19·4)	28 (90·4)	16 (69·6)	3 (13·0)	19 (82·6)	0·359	< 0·001
Febrile neutropenia	0 (0)	2 (6·5)	2 (6·5)	0 (0)	1 (3·2)	1 (3·2)	–	–
Neutropenic infection	0 (0)	1 (3·2)	1 (3·2)	0 (0)	0 (0)	0 (0)	–	1·0
Anemia	16 (51·6)	1 (3·2)	17 (54·8)	10 (43·5)	1 (4·3)	11 (47·8)	0·003	0·410
Thrombocytopenia	7 (22·6)	5 (16·1)	12 (38·7)	3 (13·0)	1 (4·3)	4 (17·3)	0·001	0·001
Non-hematological								
Vomiting	16 (51·6)	2 (6·5)	18 (58·1)	13 (56·5)	2 (8·7)	15 (65·2)	0·990	0·560
Nausea	19 (61·3)	3 (9·7)	22 (71·0)	16 (69·6)	6 (26·1)	22 (95·7)	0·029	< 0·001
Hypokalemia	3 (13·0)	1 (3·2)	4 (16·2)	6 (26·1)	2 (8·7)	8 (34·8)	0·034	0·078
Diarrhea	8 (25·8)	4 (12·9)	12 (38·7)	6 (26·1)	3 (13·0)	9 (39·1)	0·368	0·520
Constipation	6 (19·4)	0 (0)	6 (19·4)	5 (21·7)	0 (0)	5 (21·7)	0·667	–
Weight loss	10 (32·3)	0 (0)	10 (32·3)	8 (38·1)	0 (0)	8 (38·1)	0·056	–
Fatigue	20 (64·5)	0 (0)	20 (64·5)	18 (78·3)	0 (0)	18 (78·3)	0·078	–
Pain	3 (13·0)	0 (0)	3 (13·0)	2 (8·7)	0 (0)	2 (8·7)	0·580	–
Dermatitis	10 (32·3)	2 (6·5)	12 (38·8)	9 (39·1)	2 (8·7)	11 (47·8)	0·890	0·340
Urocystitis	3 (13·0)	0 (0)	3 (13·0)	1 (4·3)	0 (0)	1 (4·3)	0·085	–
Enteritis	4 (12·9)	1 (3·2)	5 (16·1)	3 (14.3)	0 (0)	3 (14·3)	0·351	0·99
Hepatotoxic event	3 (9·7)	0 (0)	3 (9·7)	2 (8.7)	0 (0)	2 (8·7)	0·830	–
Nephrotoxic event	1 (3·2)	0 (0)	1 (3·2)	2 (8.7)	0 (0)	2 (8·7)	0·089	–
Allergic reaction	2 (6·5)	0 (0)	2 (6·5)	0 (0)	0 (0)	0 (0)	0·320	–

This analysis was conducted in the safety population, which included only patients who began receiving the trial treatment. As prespecified by protocol, differences in adverse events were analyzed using χ^2^ test. For adverse events that did not meet the requirement for analysis (absolute count was 1), Fisher’s exact test was used. P value calculated with χ^2^ test.

Two patients in the nedaplatin group developed an allergic reaction and no patients had a reaction in the cisplatin group. The most frequent reasons for discontinuation of concurrent chemotherapy was thrombocytopenia in the nedaplatin group, especially in the second course during the radiotherapy. One patient died of disseminated intravascular coagulation (DIC) due to thrombocytopenia and finally respiratory failure, the reason was that this patient did not follow the doctor’s advice to regularly receive hematological and biochemical tests and timely deal with the myelosuppression after discharge from hospital.

The most frequent reasons for discontinuation of concurrent nedaplatin or cisplatin were patient refusal [2 (50%) of 4 discontinuations in the nedaplatin group vs 1 (50%) of 2 in the cisplatin group] and adverse events [1 (25%) vs 1 (50%)]. The most frequent adverse event leading to discontinuation in the nedaplatin group was thrombocytopenia. In the cisplatin group, the most frequent adverse event leading to discontinuation was neutropenia (**Appendix p4**).

### Therapy Compliance

Regarding chemotherapy compliance, as seen in **Table 4**, overall, 45/54 (83·3%) patients completed two cycles of concurrent chemotherapy, including 25/31 (80·6%) in the nedaplatin group and 20/23 (87·0%) in the cisplatin group, others had one cycle. A total of 6 (19·4%) of 31 patients received the nedaplatin dose of 80 mg/m^2^ and 25 (80·6%) received a dose of 160 mg/m^2^ in the nedaplatin group; and 3 (8·7%) of 23 patients received the cisplatin dose of 80 mg/m^2^ and 20 (87·0%) received a dose of 160 mg/m^2^ in the cisplatin group (**Appendix p5**). The survival outcomes were similar between the two dose levels (**Appendix p6**). Chemotherapy compliance did not differ between groups (*P* = 0·31, unadjusted χ² test).

**Table 4:** Compliance to concurrent chemotherapy and radiotherapy.

Variable	Nedaplatin group	Cisplatin group
Safety population	31	23
Patients receiving concurrent chemotherapy no. (%)		
Patients completing concurrent chemotherapy two cycles no. (%)	25 (80·6%)	20 (87·0%)
Patients receiving concurrent chemotherapy ≥ 160 mg/m^2^ no. (%)	25 (80·6%)	20 (87·0%)
Patients receiving EBRT no. (%)		
Patients completing RT no. (%)	29 (93·5%)	22 (95·7%)
Median (IQR) dose of CTV (Gy)	45 (22-45)	45 (18-45)
Median (IQR) dose per fraction (Gy)	1·8 (1·8-1·8)	1·8 (1·8-1·8)
Median (IQR) duration of RT (days)	37 (17-42)	37 (16-42)
Patients receiving BT no. (%)		
Patients completing RT no. (%)	26 (83·9%)	19 (82·6%)
Median (IQR) dose of EQD_2BRACHY_ (Gy)	39·7 (16-47·9)	39·7 (19·8-49·6)
Median (IQR) dose per fraction (Gy)	6·0 (6·0-7·0)	6·0 (6·0-7·0)
Median (IQR) duration of RT (days)	21 (14-35)	21 (14-35)

Regarding EBRT compliance, 29 (93·5%) of 31 patients in the nedaplatin group and 22 (95·7%) of 23 patients in the cisplatin group completed the total scheduled radiation dose. The median radiotherapy dose was 45 Gy and the median dose per fraction was 1.8 Gy. The median duration of radiotherapy was 37 days. The dose and duration of radiotherapy were well-balanced between the treatment groups.

EBRT was followed by image-guided brachytherapy (BT) with prescribed doses of 28 Gy/4 fractions or 30 Gy/5 fractions. Overall, 26 (83·9%) of 31 patients in the nedaplatin group and 19 (82·6%) of 23 patients in the cisplatin group completed the total scheduled BT dose. The median doses to the high-risk clinical target volume D90, bladder D2cc, rectum D2cc, and sigmoid colon D2cc were 84·0 Gy EQD2 (range, 58·9-105·9), 77·7 Gy EQD2 (range, 56·9-99·1), 68·0 Gy EQD2 (range, 48·6-90·7), and 62·0 Gy EQD2 (range, 39·6-83·7), respectively.

## Discussion

To our knowledge, this is the first randomized prospective trial to show, in patients with locoregional stage IIB-IVA cervical carcinoma, that nedaplatin-based concurrent chemoradiotherapy is non-inferior to standard cisplatin-based concurrent chemoradiotherapy in terms of progression-free survival and overall survival. Also, patients in the nedaplatin group had improved frequencies of hematological adverse events, but the gastrointestinal toxicities of nausea were more seen in the cisplatin group. Hepatic and nephritic adverse events were low. Our findings show that the administration of nedaplatin brought out severe hematological toxicities which led to the lower completion rate of chemotherapy, radiotherapy suspension, and even death, and eventually the pause of the trial.

Concurrent chemoradiotherapy is the standard treatment approach for patients with locoregional advanced cervical cancer. However, the use of chemoradiotherapy increases the risk of developing serious hematologic toxicity, which can impair the delivery of chemotherapy and may result in treatment interruptions. During pelvic radiation therapy, the exposure of bone marrow (BM), especially of the ilium and lumbosacral spine, remains unavoidable. A large volume of BM is irradiated, along with other critical normal tissues such as the small bowel, colon, bladder, rectum, and femoral heads, and hence the irradiation is unavoidable. Due to the high radiosensitivity of BM, radiation can induce acute and chronic pathologic and radiographic changes to the BM and lower BM activity (10, 11). Therefore, much attention should be paid to reduce the risk of therapy-associated severe events. Studies have shown the advantages of pelvic intensity-modulated radiation therapy (IMRT), including better dosimetric distribution, relatively lower irradiation dose to normal tissues, and fewer acute side effects, compared with conventional forward planning techniques (12, 13). Therefore, the bone marrow was routinely contoured to spare the patients in our study. However, myelosuppression still existed.

Based on that, the chemotherapy regimen choice has become vital. Experiences from several randomized trials found that the standard therapy for cervical carcinoma recommended by NCCN guidelines is platinum-based concurrent chemoradiotherapy, of which the most recommended is cisplatin. A retrospective study (9) found that compared with cisplatin, nedaplatin treatment expressed a higher relapse rate, lower OS, but non-significant difference in grade 3-4 toxicities. According to its observation, nedaplatin was not supported as an alternative to cisplatin in chemotherapy. While another single-arm Tohoku Gynecologic Cancer Unit study (14) demonstrated that nedaplatin-based postoperative CCRT was an effective and well-tolerated regimen for both early-stage and advanced-stage cervical cancer patients, with similar PFS, OS, and toxicities. It was efficacious and safe, with no renal toxicity for FIGO stage IB2-IVA cervical cancer (15). The same results was found in the KGROG0501 study (16).

Some alternatives are also being studied, including the regimens of nedaplatin which are used conveniently in the clinic: Platin plus 5-fluorocrail which is not recommended currently and albumin-bound paclitaxel plus platin which is currently being researched. The dose of platin in most studies was 40 mg/m^2^ every week for 5 weeks. In our study, we first used 100 mg/m^2^ every 3 weeks for two cycles, as most patients experienced chemotherapy-related adverse events, the dose was reduced to 80 mg/m^2^ because of the intolerability. So, which regimen was both effective and had low-toxicity? The randomized, prospective trial we conducted comparing cisplatin and nedaplatin was halted due to the intolerant hematological adverse events. Especially, the incidence rate of thrombocytopenia was over 35%, which mostly influenced the continuity of radiotherapy and chemotherapy.

The goal of treatment for cervical carcinoma is to improve survival and reduce toxicity. The choice of treatment regimen should be based on multiple factors, including the effectiveness of the drug and patient tolerance. Patient refusal and treatment toxicities were the most frequent reasons for discontinuation of concurrent chemotherapy. Several factors were associated with the high percentage of patient refusal. Nausea and vomiting plus hypoalimentation during chemoradiotherapy, which were mostly induced by cisplatin, led to increased fear of acute toxicities and substantially decreased patient willingness to receive the second cycle of concurrent chemotherapy. While the proportion of patients who received two cycles of concurrent chemotherapy was higher in the cisplatin group than in the nedaplatin group, most importantly, myelosuppression, which could result in death, may be the main reason for discontinuation. Also, that was why we halted the trial quickly.

Undoubtedly, the evaluation of therapeutic-associated adverse events may be influenced by various other aspects. Among them, the preventative use of recombinant human Granulocyte-Colony Stimulating Factor (rhG-CSF) selectively may more or less influence the incidence rate of granulocytopenia. Besides, the subject evaluation of gastrointestinal toxicities may more or less affect the assessment. Also, the value of the survival endpoint is limited because of the relatively small sample and short follow-up at the time of this analysis, and longer follow-up is needed to fully assess overall survival and long-term toxic effects. Further investigations are needed.

## Conclusion

Our findings suggest that nedaplatin-based concurrent chemoradiotherapy induces much more hematological toxicities in patients with locoregional advanced cervical carcinoma. The results of this trial should remind us to choose concurrent chemotherapy regimens offered to patients with cervical carcinoma cautiously.

## Data Availability Statement

The data analyzed in this study is subject to the following licenses/restrictions: The data presented in this study are available on request from the corresponding author. The data are not publicly available due to the privacy and safety. Requests to access these datasets should be directed to chenyong@mail.sysu.edu.cn.

## Ethics Statement

The studies involving human participants were reviewed and approved by The First Affiliated Hospital, Sun Yat-sen University. Written informed consent for participation was obtained when patients agreed to be enrolled in the study.

## Author Contributions

YC, YFR and XPC designed this study; SSH and YLL collected the clinical data; SSH and YW performed statistical analyses; YC and YFR gave critical comments and suggestions; SSH and YW drafted the manuscript. All authors contributed to the article and approved the submitted version.

## Conflict of Interest

The authors declare that the research was conducted in the absence of any commercial or financial relationships that could be construed as a potential conflict of interest.

## Publisher’s Note

All claims expressed in this article are solely those of the authors and do not necessarily represent those of their affiliated organizations, or those of the publisher, the editors and the reviewers. Any product that may be evaluated in this article, or claim that may be made by its manufacturer, is not guaranteed or endorsed by the publisher.
