# Supplementary material for: Concurrent Chemoradiotherapy With Nedaplatin Versus Cisplatin in Patients With Stage IIB-IVA Cervical Cancer: A Randomized Phase III Trial
Source: Front Oncol. 2022 Feb 2;11:798617. doi: 10.3389/fonc.2021.798617 (PMC8847132; doi:10.3389/fonc.2021.798617)
Supplement: Supplementary file 1 [file Presentation_1.pdf]

Supplementary Appendix

This appendix has been provided by the authors to give readers additional information about their work.

Supplement to: He S-S, Wang Y, et al. Concurrent Chemoradiotherapy With Nedaplatin Versus Cisplatin in Patients With Stage IIB-IVA Cervical Cancer: a randomised, phase 3 trial.

Appendix p1. Baseline characteristics of 54 patients in the per-protocol analysis .

Variable	N (%)	Nedaplatin Group	Cisplatin Group	*P*
Total	54 (100%)	31 (100%)	23 (100%)	
Age (years)				0·967
< 54	28 (51·9)	16 (51·6)	12 (52·2)	
≥ 54	26 (48·1)	15 (48·4)	11 (47·8)	
Stage (FIGO 2018)				0·559
II	30 (55·6)	16 (51·6)	14 (60·9)	
III	22 (40·7)	13 (41·9)	9 (39·1)	
IVA	2 (3·7)	2 (6·5)	0 (0)	
Dose of concurrent chemotherapy				
80mg/m^2	47	26	21	
100mg/m^2	7	5	2	
Death	3	2	1	
Distant metastasis	3	3	0	

Appendix p2. Disease recurrence distribution in the two treatment groups.

Variable	Nedaplatin group N=34	Cisplatin group N=34
**Disease recurrence**		
Distant	3 (%)	0
Bone	1	0
Lung	1	0
Lymph node	1	0
Death	2	1
Cancer-specific	1	1
Non-cancer-specific	1	0
Severe toxicity	1	-

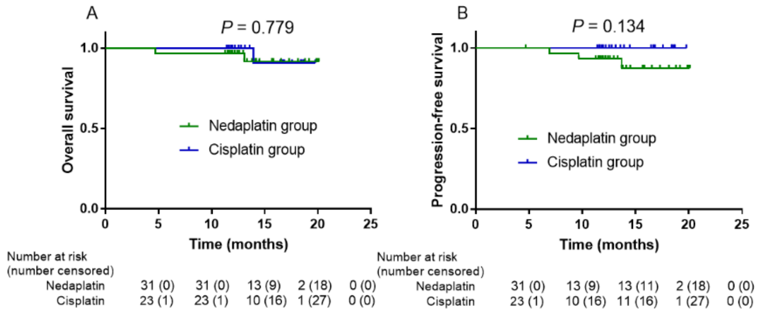

Appendix p3. In the per-protocol analysis, survival outcome differences in the nedaplatin and cisplatin groups. Kaplan–Meier overall survival (A), progression-free survival (B).

Appendix p4. The analysis of reasons for suspending concurrent chemotherapy.

Reasons	Nedaplatin group	Cisplatin group
Hematological	2 (50%)	1 (50%)
Neutropenia	1 (25%)	1 (50%)
Anemia	0	0
Thrombocytopenia	1 (25%)	0
Other toxicities	0	0
Patients' refusal	2 (50%)	1 (50%)
Accompanying disease	0	0
Others	0	0

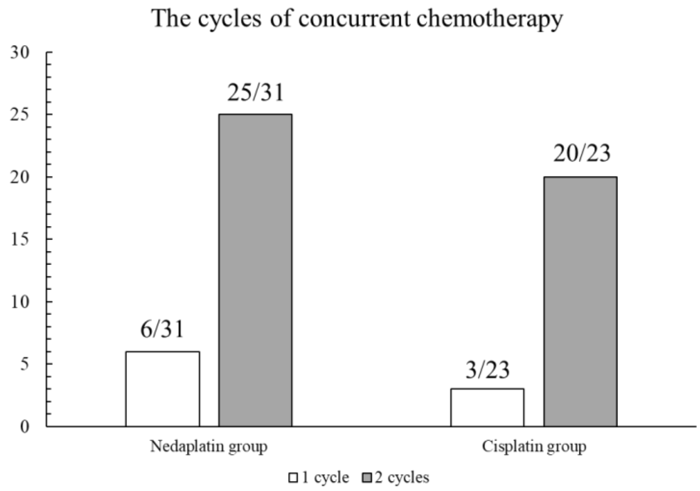

Appendix p5. The cycles of concurrent chemotherapy of nedaplatin and cisplatin group.

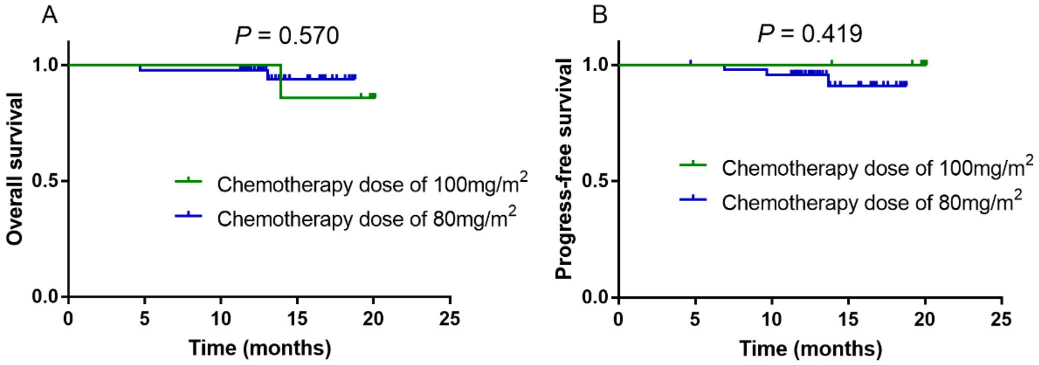

Appendix p6. Survival outcome differences in the chemotherapy dose of 100mg/m^2 and 80mg/m^2. Kaplan–Meier overall survival (A), progression-free survival (B).
